# Hormone replacement therapy prescribing in menopausal women in the UK: a descriptive study

**DOI:** 10.3399/BJGPO.2022.0126

**Published:** 2022-11-02

**Authors:** Dana Alsugeir, Li Wei, Matthew Adesuyan, Sarah Cook, Nicholas Panay, Ruth Brauer

**Affiliations:** 1 Department of Pharmacy Practice and Policy, UCL School of Pharmacy, London, UK; 2 Pharmacy Practice Department, College of Clinical Pharmacy, Imam Abdulrahman Bin Faisal University, Dammam, Saudi Arabia; 3 Department of non-communicable disease epidemiology, London School of Hygiene and Tropical Medicine, London, UK; 4 National Heart and Lung Institute, Imperial College London, London, UK; 5 Imperial College Healthcare NHS Trust and Chelsea and Westminster Hospitals, London, UK

**Keywords:** menopause, hormone replacement therapy, HRT, perimenopausal, women, primary health care, general practice

## Abstract

**Background:**

Recent studies on the prescribing of hormone replacement therapy (HRT) medicines to treat symptoms of menopause are lacking.

**Aim:**

To describe the prescribing of HRT in a cohort of UK menopausal women.

**Design & setting:**

Population-based drug utilisation study using IQVIA Medical Research Database (IMRD-UK).

**Method:**

Primary care data of women with recorded menopause and/or aged ≥50 years between January 2010 and November 2021 were extracted from the database. The incidence rate of women who received their first prescription for HRT was calculated annually using person-years-at-risk (PYAR) as the denominator. Incidence rates of HRT were estimated by type and route of administration. Relative changes in annual incidence rates were expressed as percentages and the average percentage change was assessed using linear regression. Annual prescribing prevalence per 100 women was calculated using mid-year menopausal population estimates.

**Results:**

The incidence rate of prescribing of HRT increased from 5.01 in 2010 to 18.16 per 1000 PYAR in 2021, a relative increase of 13.64% (95% confidence interval [CI] = 6.97 to 20.30) per year. The incidence rate of fixed combinations of HRT increased from 3.33 to 12.23 per 1000 PYAR in 2010 and 2021, respectively. Transdermal formulations of HRT increased from 1.48 to 14.55 per 1000 PYAR in 2010 and 2021, respectively. The overall proportion of women in receipt of a prescription for HRT changed from 7.89% in 2010 to 6.86% in 2020.

**Conclusion:**

This study shows a steady increase in the number of women receiving their first prescription for HRT during the study period, which suggests regained acceptance of HRT medicines.

## How this fits in

Recently, there has been increased societal interest in prescribing of HRT and management of symptoms of menopause. New users of HRT have markedly increased in recent years; particularly new users of non-oral formulations, and fixed combinations of oestrogen and progesterone, and younger menopausal women (aged 50–59 years). The increase in new users of symptomatic menopause treatment should inform guidance on support for GPs for prescribing of HRT and management.

## Introduction

Women going through menopausal transition typically experience symptoms starting 7 years before the last menstrual period.^1^ During menopausal transition, women commonly report genitourinary and vasomotor symptoms, sleep disturbances, cognitive decline, and mood disorders such as depression and anxiety.^2^ The National Institute for Health and Care Excellence (NICE) recommends the use of HRT to relieve symptoms of menopause with careful monitoring.^3^ HRT is composed of oestrogen and progesterone used in single form or in combination.^4^ In addition to oestrogen and progesterone, menopausal women may require testosterone supplementation for low sexual desire.^3^ There are no recent studies on the prescribing of HRT in menopausal women using patient-level data; previous studies in the UK have described prescribing rates until 2015.^5,6^ Therefore, this study aimed to describe the prescribing of HRT medicines in UK menopausal women from 2010–2021.

## Method

Anonymised patient data were extracted from IMRD-UK, which incorporates data supplied by The Health Improvement Network (THIN), a propriety database of Cegedim SA,^7^ in March 2022.

### Study population

The study population comprised menopausal women (peri- or post-menopausal) during the study period (1 January 2010–2 November 2021). Women receiving a menopausal recording before their 50^th^ birthday were included. Women with no recording of menopause (91.91%) were assumed to be menopausal from the age of 50 years onwards. This age cut-off was based on results of previous studies.^8^ In the dynamic cohort, women entered the cohort on the date of their first menopausal recording if earlier than their 50th birthday, or their 50^th^ birthday, plus a 6-month registration with the practice before the entry date. Women were censored on the earliest of the following dates: transfer out of the practice; death; or the last collection date of a primary care practice.

### Study variables

Primary care records indicating menopause were retrieved for adult women using the code list in Supplementary Table S1. Code lists for prescriptions of HRT medicines were based on *British National Formulary (BNF)* chapter 6.4.1, except for oestrogen-receptor modulators. Types of HRT were based on Anatomical Therapeutic Chemical (ATC) classification: oestrogens, progestogens, and combination regimens of oestrogen and progestin (OP) in sequential preparations or fixed combinations.^9^ Separately, prescriptions for testosterone hormone therapy were retrieved. Code lists of testosterone were based on *BNF* chapter 6.4.2 (see Supplementary Table S2 for code list). Routes of administration were grouped as the following: oral route including tablets and capsules; local route including gels, creams, and vaginal pessaries; and transdermal formulations (patches and transdermal implants).

### Statistical analysis

To describe the cohort, the proportion of menopausal recording was estimated in the following: (1) all women; and (2) women aged ≥50 years. The following variables were reported to describe the cohort: (1) age at first menopausal recording in 10-year-band age groups; and (2) HRT status.

The number of menopausal women who received their first prescription for HRT (incidence rate of prescribing) was calculated annually using PYAR as the denominator. Annual incidence rates of prescribing were estimated per 1000 PYAR with 95% CIs assuming Poisson distribution. A first prescription was defined as the first recorded prescription, with no previous recorded prescription of this medication class. Relative changes in yearly incidence rates were expressed as percentages and the average percentage change in HRT prescribing throughout the study period was assessed using linear regression. Incidence rates for prescribing of HRT were estimated separately by type of HRT, route of administration, and age group. The prescribing prevalence was calculated using mid-year population estimates as the denominator. The mid-year population was estimated by calculating the number of menopausal women on 1 July of each year. The annual prevalence was expressed per 100 women for the years with full data availability (2010–2020). Analyses were conducted using Stata (version 17).

## Results

During the study period, 1 908 177 menopausal women contributed person-years to IMRD-UK. A record of menopause was found for 8.09% (*n* = 419 755) of women of all ages (n = 5 187 388) and 18.95% (*n* = 406 516) of women aged ≥50 years (n = 2 144 861). Among women with a menopausal recording, 33.62% were aged 60–69 years. Most women with a record indicating menopause (78.27%) received prescriptions for HRT medicines (Table 1).

**Table 1.** Demographics of study cohort

Age groups^a^	Women with menopausalrecording (*n* = 419 755)	Women aged ≥50 years and/or with menopausal recording (*n* = 1 908 177)
	Women *n* (%)	Women *n* (%)
<30	401 (0.10)	401 (0.02)
30–39	2 078 (0.50)	2 078 (0.11)
40–49	26 013 (6.20)	26 013 (1.36)
50–59	127 172 (30.3)	583 679 (30.59)
60–69	141 103 (33.62)	481 214 (25.22)
70–79	93 824 (22.35)	381 102 (19.97)
≥80	29 164 (6.95)	433 690 (22.73)
HRT prescription status		
HRT Rx	328 528 (78.20)	603 013 (31.60)
No HRT Rx	91 227 (21.73)	1 305 164 (68.40)

^a^Age of women at latest date of data collection. HRT = hormone replacement therapy. Rx = prescription

### Prescribing incidence of HRT medicines

**Figure 1.**
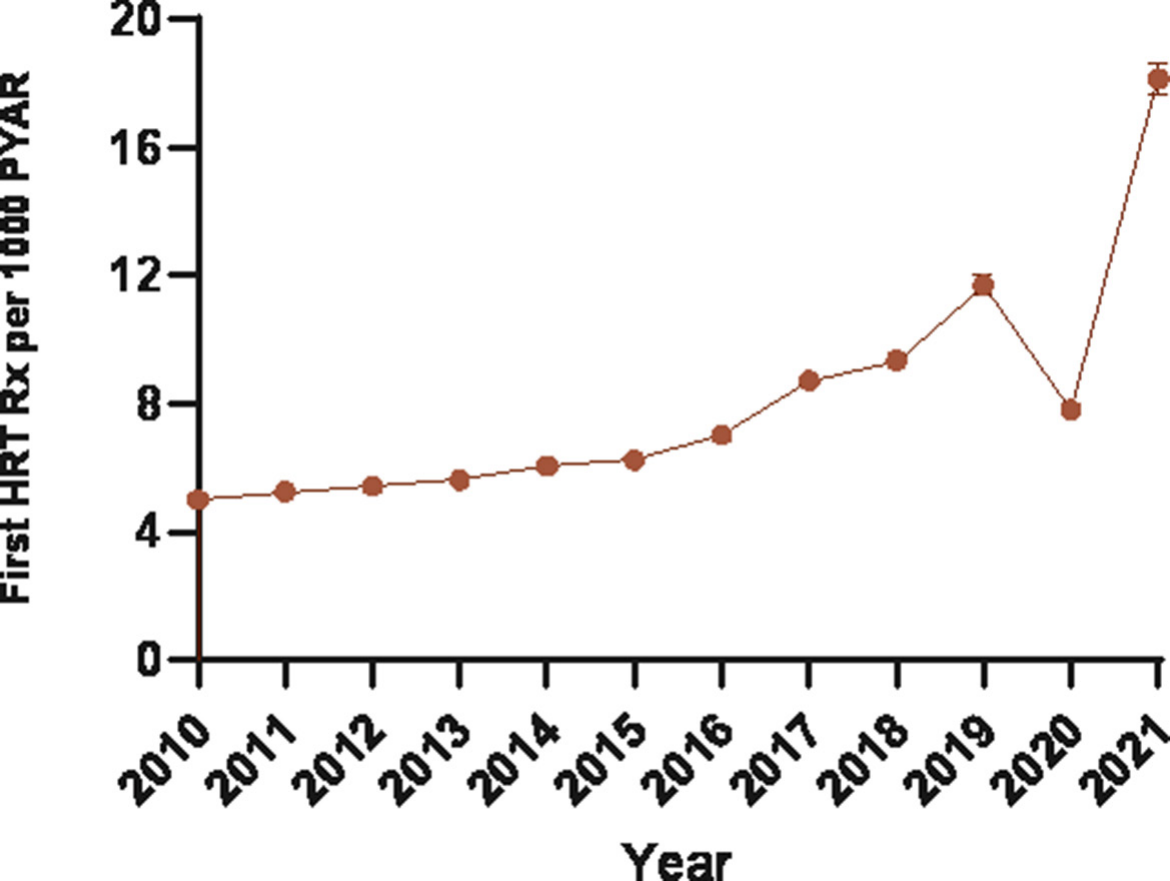
Annual incidence rate of prescribing of HRT medicines. HRT = hormone replacement therapy. PYAR = person-year-at-risk. Rx = prescription.

The incidence rate of first prescriptions for HRT medicines increased from 5.01 (95% CI = 4.87 to 5.14) in 2010 to 18.16 (95% CI = 17.66 to 18.67) per 1000 PYAR in 2021, an average annual increase of 13.64% (95% CI = 6.97 to 20.30) per year (Figure 1 and Supplementary Table S3).

**Figure 2.**
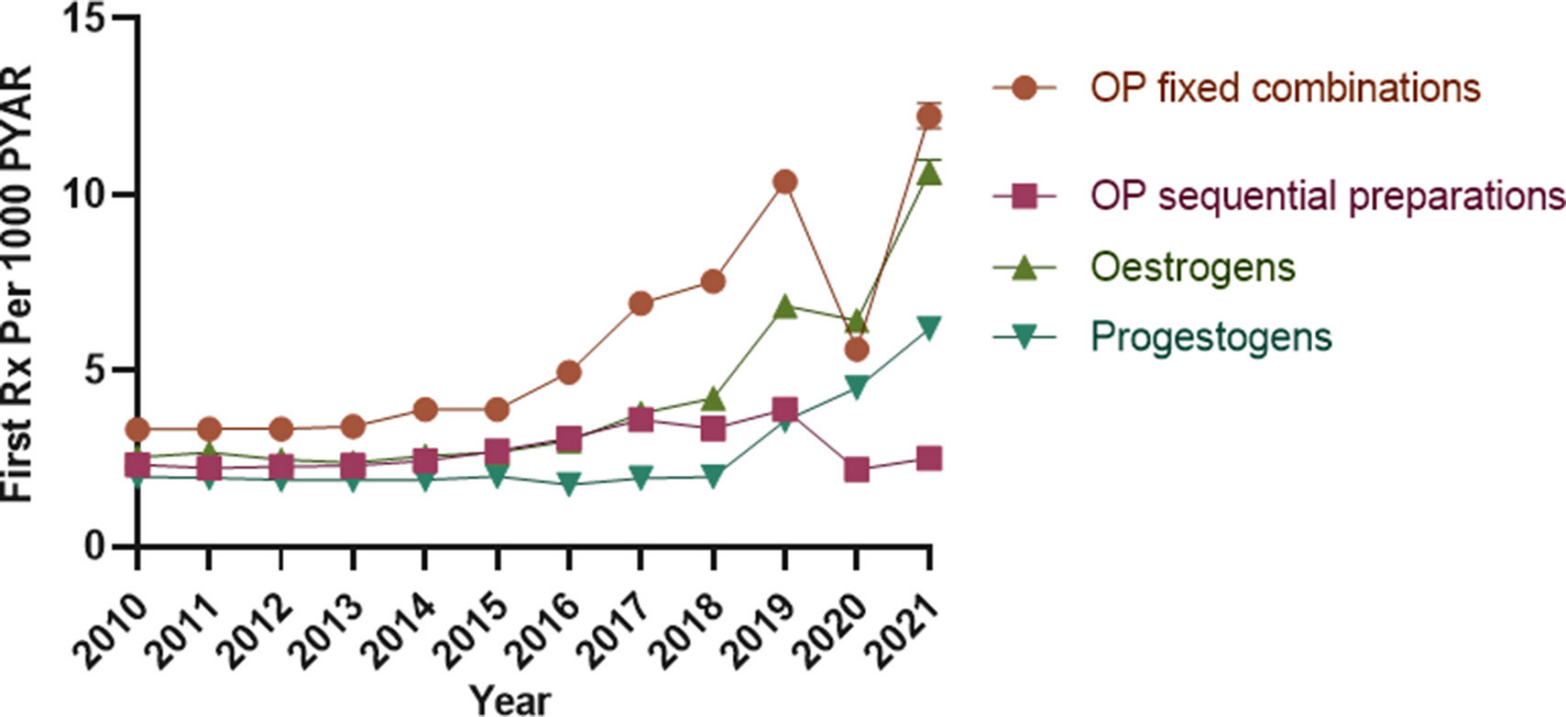
Annual incidence rate of prescribing of HRT medicines by type of HRT. HRT = hormone replacement therapy. OP = oestrogen—progesterone. PYAR = person-year-at-risk. Rx = prescription.

The highest absolute increase in new prescriptions for types of HRT was observed in fixed combinations of oestrogen and progesterone: from 3.33 per 1000 PYAR (95% CI = 3.22 to 3.45) in 2010 to 12.23 per 1000 PYAR (95% CI = 11.88 to 12.59) in 2021; an average annual increase of 17.68% (95% CI = 11.51 to 23.85). New prescriptions for oestrogen-only HRT increased from 2.53 (95% CI = 2.43 to 2.63) per 1000 PYAR in 2010 to 10.64 (95% CI = 10.30 to 10.98) per 1000 PYAR in 2021; an average annual increase of 16.70% (95% CI = 10.50 to 22.80). New prescriptions for sequential preparations of oestrogen and progesterone increased from 2.33 (95% CI = 2.23 to 2.42) per 1000 PYAR in 2010 to 2.51 (95% CI = 2.35 to 2.67) per 1000 PYAR in 2021, with a 4.39% (95% CI = 0.98 to 7.80) average annual change. Lastly, new prescriptions of progestins increased from 1.98 (95% CI = 1.90 to 2.07) per 1000 PYAR in 2010 to 6.19 (95% CI = 5.94 to 6.46) per 1000 PYAR in 2021, with an average annual increase of 13.24% (95% CI = 5.40 to 21.0) (Figure 2 and Supplementary Table S4).

**Figure 3.**
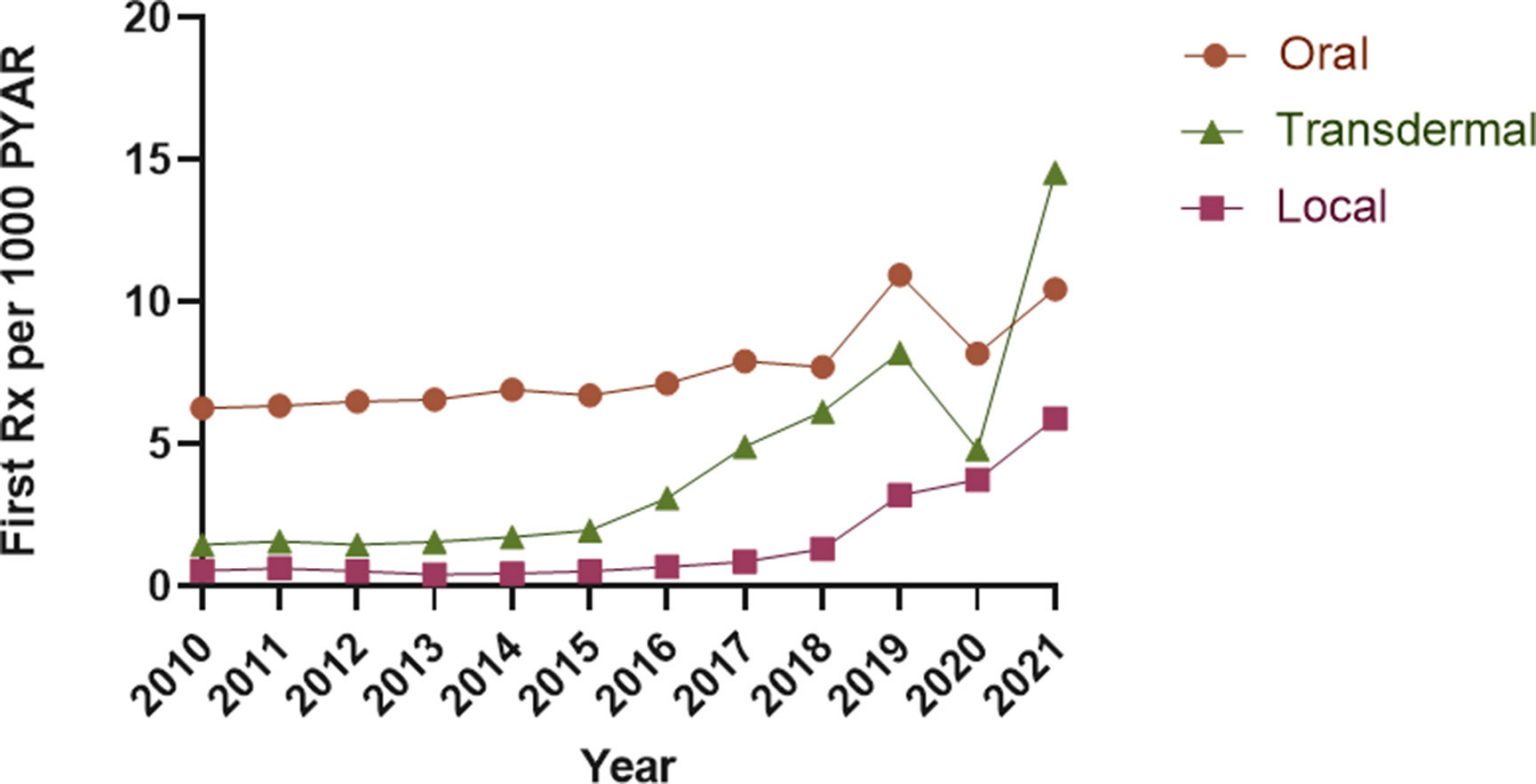
Annual incidence rate of prescribing of HRT medicines by route of administration. HRT = hormone replacement therapy. PYAR = person-year-at-risk. Rx = prescription.

Results stratified by route of administration showed an increase in all non-oral formulations of HRT. Incident prescriptions for transdermal formulations increased from 1.48 (95% CI = 1.41 to 1.55) per 1000 PYAR in 2010 to 14.55 (95% CI = 14.17 to 14.94) per 1000 PYAR in 2021, an average annual increase of 31.51% (95% CI = 20.90 to 42.12). First prescriptions for locally administered HRT increased from 0.56 (95% CI = 0.52 to 0.61) per 1000 PYAR in 2010 to 5.91 (95% CI = 5.68 to 6.16) per 1000 PYAR in 2021, or a 34.11% (95% CI = 20.67 to 47.56) average annual increase (Figure 3 and Supplementary Table S5). The prescribing incidence of oral formulations increased on average 1.67% (95% CI = –0.58 to 3.92) per study year from 6.28 (95% CI = 6.11 to 6.46) per 1000 PYAR in 2010 to 10.45 (95% CI = 10.08 to 10.84) per 1000 PYAR in 2021 (see Supplementary Table S6). The rate of first prescriptions for testosterone hormonal therapy was relatively constant during the study period, from 0.16 (95% CI = 0.14 to 0.19) in 2010 to 0.33 (95% CI = 0.28 to 0.39) per 1000 PYAR in 2021 (Supplementary Table S6).

**Figure 4.**
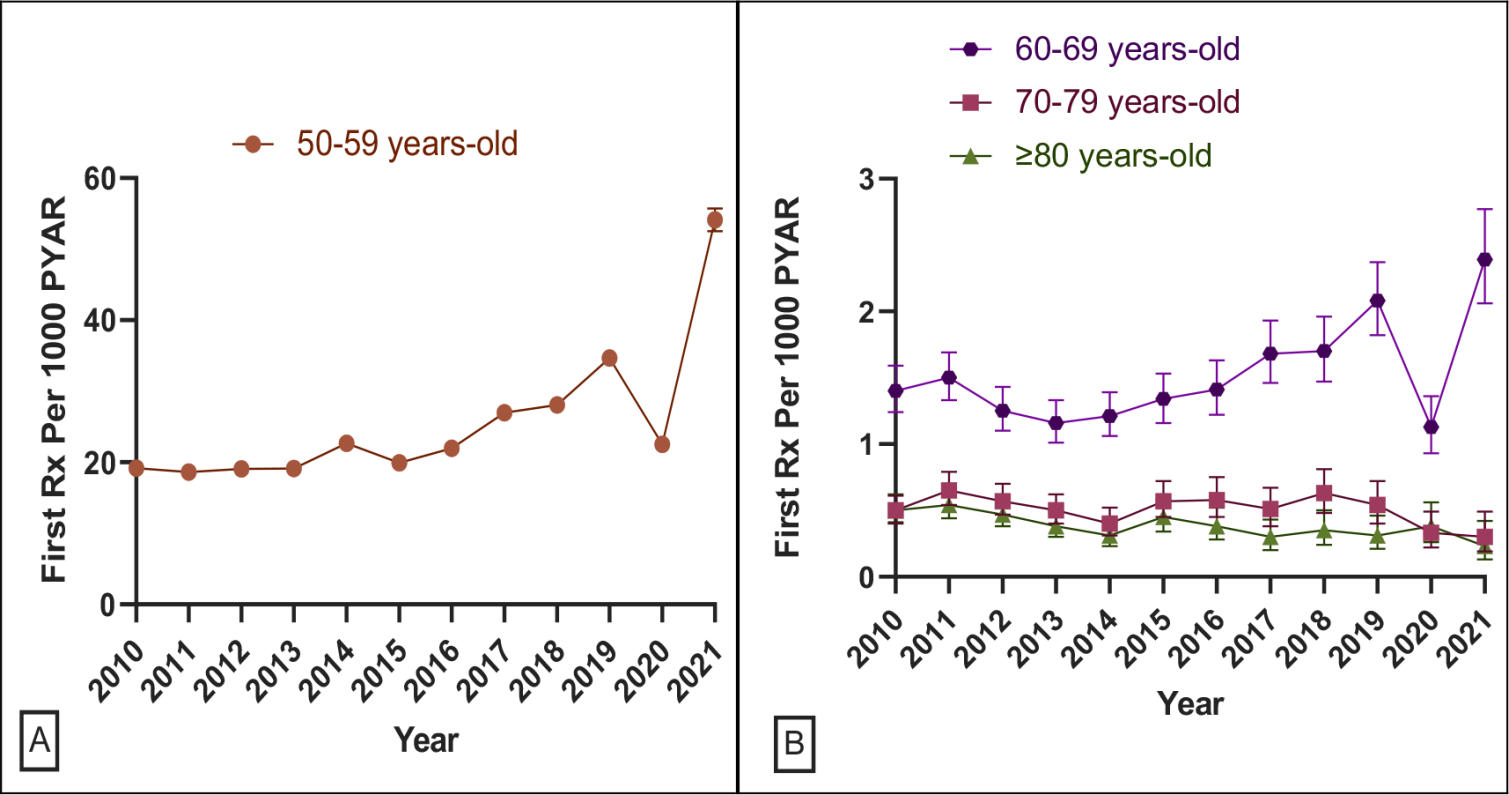
Annual incidence rate of prescribing of HRT medicines by age group. HRT = hormone replacement therapy. PYAR = person-year-at-risk. Rx = prescription.

The absolute increase in incident HRT prescribing by age was mostly observed in younger menopausal women (50–59 years). The incidence rate increased from 19.17 (95% CI = 18.6 to 19.76) per 1000 PYAR in 2010 to 54.11 (95% CI = 52.54 to 55.72) per 1000 PYAR in 2021 (see Figure 4a), an 11.71% (95% CI = 4.45 to 18.98) average annual increase. In women aged 60–69 years, first prescriptions for HRT increased on average 7.73% (95% CI = 1.72 to 13.74) per year from 2010 until 2021 (see Figure 4b and Supplementary Table S7).

### Prescribing prevalence of HRT medicines

**Figure 5.**
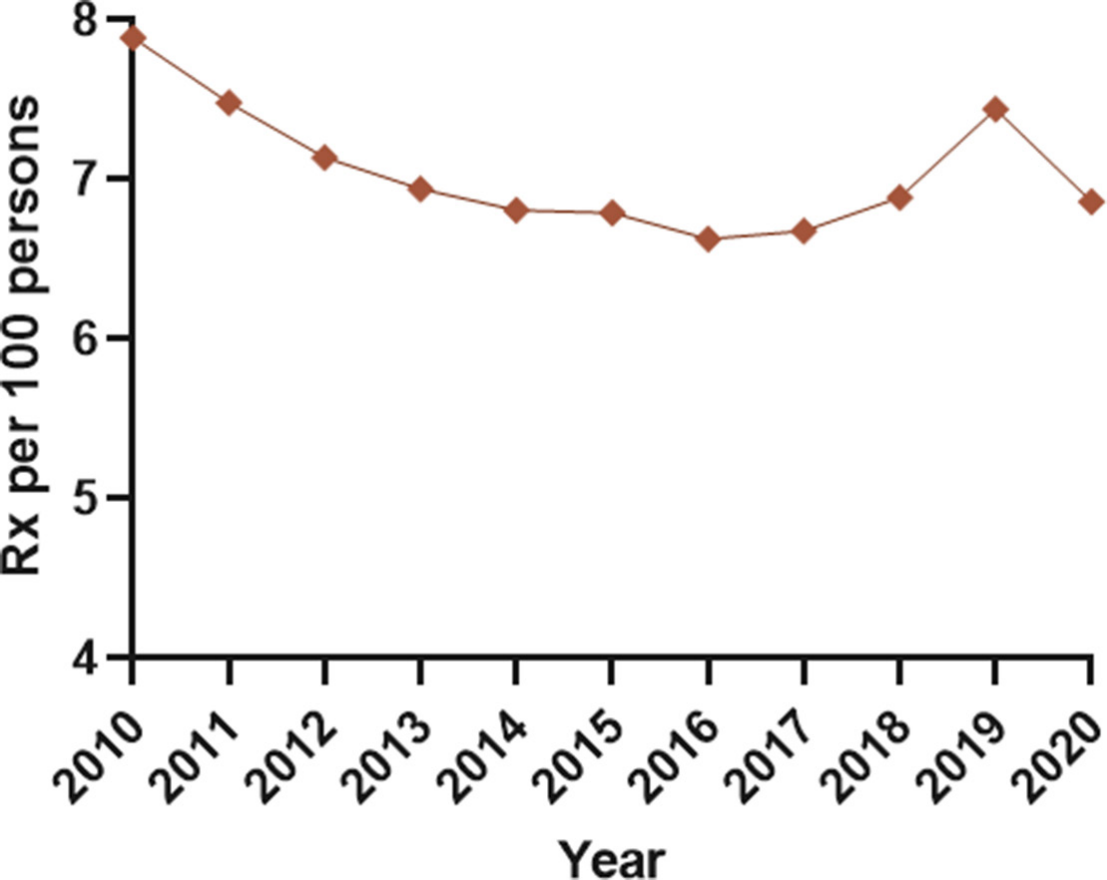
Annual prevalence of prescribing of HRT. HRT = hormone replacement therapy. Rx = prescriptions.

The annual prescribing prevalence of HRT medicines was 7.89 per 100 women in 2010 and 6.86 per 100 women in 2020, an average change of -0.38% (95% CI = -1.40 to 0.79) (Figure 5 and Supplementary Table S8).

## Discussion

### Summary

An increase was observed in new prescribing of HRT medicines from 2010–2021. The most prominent increase was seen in in the prescribing of HRT for younger women (50–59 years), non-oral routes of administration, and fixed combinations of oestrogen and progesterone.

### Strengths and limitations

Recently, the "all party parliamentary group on menopause" recommended further studies on how medical professionals treat menopause^10^. This study provides up-to-date information on the prescribing of HRT in the UK using patient-level data. Moreover, the study is the first to report on the prescribing of testosterone in menopausal women. Menopausal onset is not well recorded in primary care databases, thus it was assumed that women were menopausal on or after the age of 50 years. Some women may have had their menopausal onset before or after the age of 50 years, and therefore the incidence and prevalence of prescribing of HRT may have been underestimated. An underestimation of prescribing may have also occurred as some women may be treated in specialist centres and prescriptions issued outside primary care practices are not recorded in the IMRD-UK database. In addition, IMRD-UK has a unique patient identifier per practice. Thus, women switching practices would be identified as receiving new prescriptions rather than repeat prescriptions. Further, indications for medicine use are not recorded in IMRD-UK database. Therefore, medicines such as progestins and sequential hormone preparations may have been prescribed for other conditions than menopause management. Lastly, the IMRD-UK database only has information on the prescribing of medications, but information on whether prescriptions were redeemed or consumed is not available.

### Comparison with existing literature

Our results update previous reports that described the prescribing of HRT in the UK.^5,6^ Prescribing rates of HRT medicines have been affected by early results of the Women's Health Initiative (WHI) trial.^11,12^ Following the trial, prescribing of HRT in the UK decreased between 2000 and 2005 and then remained relatively constant until 2015.^5,13^ Similar trends have been observed internationally.^14–16^ The present study found a rapid increase in incident prescribing of HRT from 2015 onwards, except for the year 2020. A decrease in prescribing in 2020 may be a result of the decline in healthcare utilisation owing to restrictions imposed during the COVID-19 pandemic.^17^ As 2020 was the last full year of data collection, this also affected the estimates of annual change in the prescribing prevalence. The increase in incident prescribing of HRT from 2015 onwards could be explained by the publication of the NICE guidelines for management of menopause,^3^ and publication of the sub-group analyses of the WHI trial.^12,18^ The increase in prescribing may also be driven by raised awareness of menopause.^19^ Several menopause support organisations are raising awareness on menopause issues and educating healthcare professionals on menopause management.^19–21^ Since the first publication of the initial WHI, more research has been conducted on different formulations and types of HRT medicines.^22^ Studies have shown that non-oral formulations of HRT are associated with a lower risk of venous thromboembolism (VTE) compared with oral formulations of HRT.^23–25^ In the present study, the rise in new prescriptions of HRT medicines in non-oral formulations was marked. In addition, a 17.68% relative increase was observed in prescribing of fixed combinations of OP compared with 4.39% increase in sequential preparations. This could be in response to safety reports indicating that progesterone used in sequential combinations, such as medroxyprogesterone acetate, are associated with higher VTE risk.^25,26^ Testosterone is indicated for women suffering from hypoactive sexual desire disorder (HSDD) as a second-line treatment after HRT.^3^ Use of testosterone replacement was limited in the present study as testosterone formulations are only available in male doses through specialist prescribing.^27^

The results of the sub-group analyses of WHI suggest that HRT is not associated with increased risk of cardiovascular disease if started soon after menopause or in young menopausal women, generating the 'timing hypothesis'.^3,12,26,28^ In the present study, the absolute rates of new prescribing of HRT were highest (11.71% per year) in women aged 50–59 years throughout the study period. These results are consistent with previous reports using UK primary care data, in which the prevalence of HRT prescribing up to 2016 was higher in women aged 50–59 years.^5,13^ However, the relative increase in new prescribing of HRT in women aged 60–69 years during the study period was 7.73% per year, showing that the increase in HRT prescribing is not just driven by the youngest menopausal age group.

The study shows that the overall proportion of women who received a prescription for HRT, both new users and prevalent users, increased until 2019, but that the increase was highest for new prescriptions. This suggests that clinical guidelines were followed and HRT is used for the short-term management of menopausal symptoms.^3^ In women with early menopause, HRT is recommended until they reach the age of natural menopause.^3^ However, it may not have affected the prescribing prevalence of HRT in the present study as a small proportion of the cohort were recorded as ‘early menopausal’. The study shows that menopausal onset is not well recorded in primary care records. Only 18.95% of women aged ≥50 years had a record of menopause. Therefore, it is hard to infer menopausal onset from the study.

### Implications for practice

Increased prescribing of medicines used for the management of symptoms of menopause may reflect improved awareness by women and healthcare professionals. The findings suggest that the initial decrease in HRT prescribing after the first WHI trial has recovered. HRT therapy offers holistic management for menopausal symptoms, in addition to preventing osteoporosis and improving quality of life.^29^ Given the recent increase in HRT prescribing, the authors support the recommendation of the British Menopause Society that all healthcare professionals should have a basic understanding of menopause, in addition to including a GP with special interest in menopause in each primary care team.^30^ To improve understanding of menopause and its management in the UK primary care setting, the authors recommend enhancing recording of natural and medical causes of menopause by GPs. Furthermore, the recommendation of NICE guidelines to review and weigh the risk or benefits of HRT treatment regularly is supported.^3^

## Data

IQVIA Medical Research Data (IMRD) incorporates data from THIN, A Cegedim Database. Reference made to THIN is intended to be descriptive of the data asset licensed by IQVIA. IMRD data governance does not allow us to distribute patient data to other parties. Researchers may apply for data access at https://www.iqvia.com/library/fact-sheets/uk-emr-iqvia-medical-research-data. The codes used to produce the data for this study are provided in the Supporting Information.
