# Correction: Hormone replacement therapy prescribing in menopausal women in the UK: a descriptive study

**DOI:** 10.3399/BJGPO.2022.0126.C1

**Published:** 2022-12-31

In the article by Alsugeir *et al*, Hormone replacement therapy prescribing in menopausal women in the UK: a descriptive study. BJGP Open 2022; DOI: https://doi.org/10.3399/BJGPO.2022.0126, there was an omission from the competing interests paragraph. The text should read: ‘Nicholas Panay is the founder of a private menopause clinic and has received funds for research and speaking from pharmaceutical companies. He was not involved in the analysis of this study. The other authors have no competing interests.’ The online version has been corrected.

